# Health-related quality of life, work ability and disability among individuals with persistent post-dural puncture headache

**DOI:** 10.1186/s10194-024-01765-8

**Published:** 2024-04-24

**Authors:** Ali Kapan, Thomas Waldhör, Tobias Schiffler, Jürgen Beck, Christian Wöber

**Affiliations:** 1 Center for Public Health, Department of Social and Preventive Medicine, Medical University of Vienna, Vienna, Austria; 2 Center for Public Health, Department of Epidemiology, Medical University of Vienna, Vienna, Austria; 3 Department of Neurosurgery, University Medical Center Freiburg, Freiburg, Germany; 4 Department of Neurology, Medical University of Vienna, Vienna, Austria; 5 Comprehensive Center for Clinical Neurosciences and Mental Health, Medical University of Vienna, Vienna, Austria

**Keywords:** Postdural puncture headache, Cerebrospinal fluid leak, Persistent pain, Disabilty, Quality of life

## Abstract

**Background and objectives:**

Postdural puncture headache (PDPH) is an acknowledged consequence of procedures like lumbar punctures, epidural analgesia, and neurosurgical interventions. Persistence over more than three months, however has been poorly studied. In particular, little is known about the impact of persistent PDPH (pPDPH) on health related quality of life (HRQoL), disability and ability to work. The study aimed to provide a holistic understanding of pPDPH, encompassing medical, physical and psychological aspects.

**Methods:**

We conducted a cross-sectional anonymous online survey in individuals aged 18 or older, diagnosed with, or suspected to have pPDPH via self-help groups on Facebook. Participants completed a structured questionnaire covering diagnosis, symptoms, and the ability to work. For assessing headache related disability, and mental health, they filled in the Henry Ford Hospital Headache Disability Inventory (HDI) and the Depression Anxiety Stress Scale-21 (DASS-21).

**Results:**

A total of 179 participants (83.2% female, mean age 39.7 years) completed the survey. PPDPH had been present for one year or more in 74.3%, and 44.1% were unable to be in an upright position for more than one hour per day without having to lie down or sit down. Headaches were extremely severe or severe in 18% and 34%, respectively. According to the HDI, 31.8% of participants had mild, 25.7% moderate, and 42.5% severe disability. DASS-21 revealed substantial mental health challenges with depression, anxiety and stress experienced by 83%, 98%, and 88% of the respondents. The ability to work was limited considerably: 27.9% were unable to work, 59.8% worked part-time, 1.1% changed their job because of pPDPH, and only 11.2% were able to work full-time in their previous job. Despite treatment, the patients’ condition had deteriorated in 32.4% and remained unchanged in 27.9%.

**Conclusion:**

This study stresses the burden of pPDPH in terms of substantial disability, limited quality of life, mental health concerns, and significant impact on the ability to work. The study highlights the long-term impact of pPDPH on individuals, emphasizing the need for timely diagnosis and effective treatment. It underscores the complexity of managing pPDPH and calls for further research into its long-term effects on patient health and HRQoL.

**Supplementary Information:**

The online version contains supplementary material available at 10.1186/s10194-024-01765-8.

## Background

Postdural puncture headache (PDPH) is a well-known adverse effect that can occur after different medical procedures such as lumbar puncture, epidural analgesia, and neurosurgical procedures [1, 2]. PDPH typically develops within five days and is characterized by worsening of the headache with standing or sitting, which improves when lying flat [3]. The theorized cause behind PDPH is the leakage of cerebrospinal fluid (CSF) following a dural puncture, which results in the “sagging” of intracranial structures and consequent intracranial hypotension (IH), as well as cerebral and meningeal vasodilation [4]. Most patients experience symptoms relief within four days, and approximately 85% recover within six weeks with conservative treatment such as rest, hydration, analgesics, and caffeine intake. If conservative measures remain ineffective, epidural blood patch (EBP) is recommended [5]. However, its efficacy, actually varies from 55 to 75%, depending on the specific cause of the dural pathology and the conditions of its application performed [6, 7]. Moreover, even if clinical improvement occurs in the early follow-up period, persisting CSF loss may perpetuate the disease and potentially result in severe long-term morbidity [8, 9]. Notably, the persistent form of PDPH (pPDPH), while clinically observed [7, 9], is not formally recognized in the International Classification of Headache Disorders 3rd Edition (ICHD-3). This oversight highlights a gap in the current understanding and classification of pPDPH underscoring the need for further research into its prolonged manifestations. The understanding of the physiological basis for pPDPH remains elusive [9, 10]. It is increasingly suggested that the persistent headache could be ascribed to either an unaddressed dural defect [11], resulting in ongoing CSF leakage, or to a central sensitization phenomenon triggering persistent headaches even after the healing of the dural defect [12]. The latter notion is underscored by the findings of Ansari et al. [10], who observed a significant number of control group patients experiencing orthostatic headaches in the absence of dural rent [7]. Studies, such as a multicentre study involving 25 patients who underwent contrast-enhanced brain MRI [12], and another by Schievink et al. [13], retrospective case series, underscores the diagnostic challenges of this condition. In this study, only a small fraction of patients with pPDPH exhibited typical low intracranial pressure signs on brain MRI, suggesting that PDPH can occur without significant IH or the expected radiological signs. Moreover, digital subtraction myelography localized leaks in patients with identified leaks on spinal MRI or CT myelography and diagnosed a cerebrospinal fluid-venous fistula in a patient with normal spinal imaging. These findings highlight the complex pathophysiology of pPDPH, indicating that it may not always align with traditional concepts of IH, emphasizing the importance of a comprehensive diagnostic evaluation.

Consequently, many patients often undergo multiple examinations over an extended duration until a definitive diagnosis is established [14]. While specific data on pPDPH is limited to case studies [15], research into Spontaneous Intracranial Hypotension (SIH) indicates that delays in treatment can adversely affect recovery chances [16, 17]. In particular, early treatment within 12 weeks is associated with better outcomes [18] highlighting the critical importance of rapid diagnosis and intervention in conditions with similar symptomatology to PDPH, like SIH. The potentially irreversible and severe morbidity associated with long-standing CSF leakage with the development of persistent headaches and various additional symptoms (e.g. including neck pain, neck stiffness, nausea, vomiting, tinnitus, diplopia, brain fog, cognitive deficits and decreased level of consciousness) can lead to significant disability-including lost workdays, unemployment, disability, and changes in their health-related quality of life (HRQOL).

In the realm of medical research concerning PDPH, there is a recognized need for a comprehensive examination of its persistent form, pPDPH, and the diverse symptoms that may accompany this condition [15]. Surprisingly, despite the incidence of persistent headaches in accidental dural puncture patients being as high as 38%, these cases are seldom seen in pain clinics [10, 12, 19, 20]. This discrepancy highlights a gap in the existing literature, which has not thoroughly investigated the impact of pPDPH on aspects such as patients’ social life, work ability, and HRQoL. This study aims to address this deficiency by conducting an extensive online survey targeting individuals with a diagnosis or suspected pPHPD. The primary objective is to understand not only the direct effects of pPDPH but also how it may influence various aspects of patients’ lives. By providing this comprehensive perspective, the study hopes to offer guidance that healthcare professionals and insurance providers can use to make more informed decisions concerning diagnosis, treatment, and support for those affected by this condition.

## Methods

### Settings and participants

This study, structured as a global online cross-sectional survey, was conducted over eight weeks from 19. April to 19. June 2023. Ethical approval was procured from the Ethics Committee of the Medical University of Vienna, under the approval number 1180/2023. Before participation, individuals were required to provide their consent, which entailed clicking on the “Agree” box. This action signified their understanding of the participant information sheet and their confirmation of meeting the inclusion criteria. For distribution, the survey link was disseminated through various organizations’ social media platforms, including Facebook, Twitter, and Instagram. These organizations comprised spinalcsfleakcanada.ca, csfleak.uk, Liquorunterdrucksyndrom.de, Lumbar Puncture Leakers, and CSF Leaks International. Cumulatively, these platforms have a reach exceeding 17.000 followers. It is pertinent to note the potential for overlap, as some individuals might follow multiple pages. To ensure continuous engagement, weekly reminders about the survey were periodically posted on these platforms.

The inclusion criteria for our study mandated that participants be at least 18 years old. Enrolment was specifically limited to individuals who affirmatively responded to the question, “If you have been diagnosed with PDPH, we would be very grateful if you could complete this questionnaire to the best of your knowledge and belief”. Eligibility was contingent upon experiencing pain for more than three months. Moreover, inclusion required participants to have received at least one EBP, possess the ability to read and understand English, and have given their consent to participate. Individuals with a known history of spontaneous intracranial hypotension (SIH) were excluded to ensure the study focused solely on participants affected by PDPH.

### Survey development

The design of this study, a close collaboration between clinical researchers and pPDPH patients emphasizing a patient-centered approach, began with ensuring that all data collected reflected participants’ experiences and medical examinations up to the start of our survey. The active involvement of an author who has personal experiences with pPDPH further provided invaluable insights, significantly enriching the survey’s content. In terms of data collection, comprehensive socio-demographic details were sought, including age, gender, educational level, and professional status. The survey’s design incorporated a mix of open-ended, quantitative, and multiple-choice questions to thoroughly address the multifaceted aspects of pPDPH, ranging from symptoms to diagnostic modalities and treatments. Moreover, the survey concentrated on various medical interventions. This encompassed the treatments administered, their duration, and the consequent outcomes. It was supplemented with information about pharmaceutical treatments, psychological therapies, and related hospitalizations, while the diagnostic modalities and treatments were published elsewhere. Beyond disease-specific questions, standardized evaluation tools were incorporated to offer a holistic perspective on the general well-being of participants. Notably, the survey was designed to capture experiences and interventions occurring up to the point of survey completion, ensuring that the data reflects the participants’ history up to and including events before the survey. The publicly accessible online questionnaire was designed in accordance with the Checklist for Reporting Results of Internet E-Surveys (CHERRIES) [21] guidelines using https://www.soscisurvey.de.

### Questionnaires

#### The depression anxiety stress scale-21 (DASS-21)

The DASS-21 is a condensed instrument that effectively measures three key mental health constructs: depression, anxiety, and stress. Suitable for both clinical and non-clinical adult populations, the DASS-21 is widely recognized for its validity in evaluating these adverse psychological states. The DASS-21 questionnaire comprises 21 items, organized into three self-report scales, each targeting a specific construct - depression, anxiety, or stress. Each scale contains seven distinct elements, evaluated on a four-point Likert scale. The scoring is as follows: 0 for “Did not apply to me at all,” 1 for “Applied to me to some degree or some of the time,” 2 for “Applied to me to a considerable degree or a good part of the time,” and 3 for “Applied to me very much or most of the time.” To compute the scores for depression, anxiety, and stress, the corresponding items’ scores are summed. As the DASS-21 is a shortened version of the original DASS, which contains 42 items, it is necessary to multiply each subscale score by two to yield the final score. These scores are then categorized as per the DASS manual into the following categories: “normal,” “mild,” “moderate,” “severe,” or “extremely severe.” This standardized scoring and classification system allows for the accurate and consistent measurement of these three crucial psychological states, contributing to its broad adoption in mental health assessment [22, 23].

#### The Henry Ford hospital headache disability inventory (HDI)

The HDI is a reliable and widely accepted tool used to assess the severity and frequency of headaches, as well as their impact on daily life. It is particularly effective for evaluating the effectiveness of non-pharmacological interventions in individuals experiencing frequent episodic or chronic migraines, aligning with the endpoints recommended by the International Headache Society. The HDI consists of 25 items, split into two subscales: a functional subscale comprised of 12 items (assessing functional disability) and an emotional subscale consisting of 13 items (evaluating emotional disability). Each item offers three possible responses: “no” (equating to 0 points), “sometimes” (2 points), and “yes” (4 points). The total score, or overall disability, is derived from summing these points, with possible scores ranging from 0, indicating no disability, to 100, representing maximum disability. The cut-offs for the HDI scores are: Mild Disability (0–30 points), Moderate Disability (31–60 points), and Severe Disability (> 60 points) [24].

### Statistical analysis

The statistical analysis of our study differentiated between two specific groups: the confirmed group and the unconfirmed group. The confirmed group consisted of individuals who had evidence of a CSF leak or IH in radiological imaging. In contrast, the unconfirmed group included individuals without these specific radiological findings. To ensure the integrity of this classification, only participants who had undergone at least one MRI examination of the spine as well as the brain were included for analysis. In the analysis, continuous measures were reported as means and standard deviations, and categorical variables were presented as frequencies and percentages.

In our study, we conducted several binary logistic regressions to investigate the relationship between confirmed and unconfirmed PDPH as the dependent variable, with various health and psychological variables as independent variables. These included depression, stress, level of disability, headache intensity, the orthostatic component of headaches (yes/no), and experiences of trauma (DASS-21 anxiety was not considered as a variable in our analysis, as all but one participant reported experiencing anxiety, making it a near-constant factor across the sample). To maintain the integrity of the analyses and ensure a homogeneous sample and clear inference, we excluded patients who underwent surgery from our analysis. This exclusion was to prevent increased variance and potential distortion of the effects of non-surgical treatments and to avoid separation issues, as all had confirmed diagnoses. Covariates in the regressions included age, gender, duration of PDPH, presence of headaches prior to the onset of PDPH, and the number of EBP performed. These were incorporated to gain a comprehensive understanding of the factors potentially associated with the diagnostic status of PDPH.

Furthermore, we employed a generalized linear model using a Poisson distribution with a log-link function to with the count of EBPs as dependent variable. As independent variables, we selected gender, age, the presence of headache before the onset of PDPH, and the duration of PDPH. After controlling for potential confounding factors, including gender, age, headache before the onset of PDPH, and the duration of PDPH, we incrementally introduced various health and psychological variables into the core model. These variables encompassed depression, stress, level of disability, headache intensity, the presence of orthostatic components in headaches (yes/no), and experiences of trauma. Furthermore, we examined an interaction effect between gender (male or female) and the headache before the onset of PDPH (present or absent) within the model to account for potential gender-specific disparities and comorbidities. All analyses aimed to accurately and comprehensively represent the collected data and were performed using SPSS software version 27. P-values were not adjusted by the number of tests and therefore p-values should be interpreted as exploratory only.

## Results

All findings reported subsequently rely exclusively on the participants’ report.

### Participant demographics

The survey received a total of 1,448 clicks, including accidental double clicks and views from search engines. Of these, 347 individuals agreed to participate in the survey. Out of these participants, 86 indicated they experienced SIH, whereas 37 individuals indicated that they had neither been suspected of nor diagnosed with PDPH by a physician. Forty-five participants withdrew from the study before completing the questionnaire, having only filled out the demographic sections. Finally, 179 participants diligently finished the questionnaire, thereby meeting the inclusion criteria for the study. Two different groups were distinguished: the confirmed group (*N* = 82) and the unconfirmed group (*N* = 97). The confirmed group comprised individuals in whom radiological imaging had identified a CSF leak or signs of IH, whereas individuals in the unconfirmed group did not exhibit these specific findings. The mean age of the participants was 39.7 ± 9.1 years, 149 (83.2%) were female. With respect to educational and professional backgrounds, roughly one third were in training, employed/self-employed or disabled/unable to work (Table 1). Furthermore, nearly 10% of the participants reported having Ehlers-Danlos Syndrome, and slightly over 5% indicated they have Postural Orthostatic Tachycardia Syndrome (Appendix Table 1).

**Table 1:** Demographic Characteristics of Participants

Variables	Total *N* = 179	Confirmed *N* = 82	Unconfirmed *N* = 97
	*N* (%)	*N* (% )	*N* (%)
Gender			
Female	149 (83.2)	70 (85.4)	79 (81.4)
Male	30 (16.8)	12 (14.6)	18 (18.6)
Age Mean (SD) min-max	39.5 (8.9) 20–77	38.3 (9.5) 20–75	40.6 (8.3) 20–77
Marital Status			
Married	61 (34.1)	23 (28.0)	38 (39.2)
Divorced	12 (6.7)	2 (2.4)	10 (10.3)
Single	46 (25.7)	23 (28.0)	23 (23.7)
Widowed	1 (0.6)	1 (1.2)	0 (0.0)
In a Relationship	59 (32.9)	33 (40.2)	26 (26.8)
Education			
Training/Apprenticeship	33 (18.4)	23 (28.0)	10 (10.3)
University Student	22 (12.3)	9 (11.0)	13 (13.4)
Employee	44 (24.6)	19 (23.2)	25 (25.8)
Self-employed	23 (12.8)	6 (7.3)	17 (17.5)
Unemployed/Seeking Employment	1 (0.6)	0 (0.0)	1 (1.0)
Prefer not to answer	2 (1.1)	1 (1.2)	1 (1.0)
Disabled/Unable to Work	54 (30.2)	24 (29.3)	30 (30.9)

### Contributing factors and antecedent headache preceding the onset of PDPH

Forty participants (22.3%) reported experiencing headaches in the three months preceding the onset of PDPH. Of these, migraine was more prevalent in the ‘Confirmed’ group, affecting 50%, compared to 16.7% in the ‘Unconfirmed’ group. Tension headaches were similarly distributed across both groups. The primary cause of PDPH, in nearly half of the cases across the entire group, was lumbar puncture for cerebrospinal fluid examination. About a quarter of the cases were due to epidural anaesthesia, and around 17% were linked to epidural injections. Less common causes included lumbar punctures for imaging procedures and spinal surgery. Suspicion of PDPH within one day occurred in nearly 20% of the confirmed group, compared to 6.2% of the unconfirmed group. Furthermore, almost a quarter of the ‘Unconfirmed’ group raised suspicion in less than a week, against 4.9% in the ‘Confirmed’ group. At three months, the rates of suspected and diagnosed PDPH had risen to 70.4% and 56.4%, respectively, across the entire group (Table 2).

**Table 2:** Pre-Existing Headaches, Contributing Factors and Diagnosis Timelines

Variables	Total *N* = 179	Confirmed *N* = 82	Unconfirmed *N* = 97
	*N* (%)	*N* (%)	*N* (%)
Headache before onset of PDPH			
Yes	40 (22.3)	22 (26.8)	18 (18.6)
No	139 (77.7)	60 (73.2)	79 (81.4)
Type of Headache before onset of PDPH			
Migraine	14 (35.0)	11 (50.0)	3 (16.7)
Tension Headache	17 (42.5)	8 (36.4)	9 (50.0)
No Physician Consultation	9 (22.5)	3 (13.6)	6 (33.4)
Frequency of Headache before onset of PDPH			
Less than one day per month	22 (55.0)	10 (45.5)	12 (66.7)
One to 14 days per month	16 (40.0)	11 (50.0)	5 (27.8)
15 or more days per month	2 (5.0)	1 (4.5)	1 (5.6)
PDPH Attributed to			
Lumbar puncture for CSF examination	88 (49.2)	44 (53.7)	44 (45.4)
Lumbar puncture for imaging procedures	8 (4.5)	6 (7.3)	2 (2.1)
Epidural Anaesthesia	41 (22.9)	11 (13.4)	30 (30.9)
Epidural Injections	30 (16.8)	12 (14.6)	18 (18.6)
Spinal Surgery	12 (6.7)	9 (11.0)	3 (3.1)
Time Before Suspicion			
Less than one day	21 (11.7)	15 (18.3)	6 (6.2)
Less than one week	27 (15.1)	4 (4.9)	23 (23.7)
1 to 4 weeks	27 (15.1)	15 (18.3)	12 (12.4)
1 to 3 months	51 (28.5)	29 (35.4)	22 (22.7)
More than three months	53 (29.6)	19 (23.2)	34 (35.1)
Time Before Diagnosis			
Less than one day	15 (8.4)	9 (11.0)	6 (6.2)
Less than one week	20 (11.2)	6 (7.3)	14 (14.4)
1 to 4 weeks	12 (6.7)	5 (6.1)	7 (7.2)
1 to 3 months	32 (17.9)	20 (24.4)	12 (12.4)
More than three months	100 (55.9)	42 (51.2)	58 (59.8)

### Characteristics of duration, disability, and work capacity in pPDPH

At the time of the survey, slightly more than 40% of the respondents reported suffering from persistent headaches following perforation for 1 to 3 years, with one-third of the entire group experiencing this condition for three or more years. Notably, around a third of confirmed cases had headaches for 3–5 years, while it was just above 10% for unconfirmed cases. In terms of disability in the total group, about a third reported mild, a quarter moderate, and nearly two-fifths severe disability. Among confirmed cases, almost half had mild disability compared to almost a fifth in the unconfirmed group. Conversely, over a third in the unconfirmed group had moderate disability, compared to nearly 15% in the confirmed group. Overall, slightly more than half experienced positional headaches, with a higher percentage in the unconfirmed group (62.9%) compared to the confirmed group (45.1%). Regarding the ability to stay upright, nearly one third could remain upright for less than an hour. However, the confirmed group had a higher percentage (43.9%) who could stay upright all day, while it was 19.6% in the unconfirmed group. In terms of workability, 17.1% in the confirmed group could work full-time compared to 7.2% in the unconfirmed group. Nonetheless, similar proportions (about 27%) in both groups were unable to work due to their condition, and the majority in both confirmed (54.9%) and unconfirmed (62.9%) cases could only work part-time. Further details about these restrictions can be found in Table 3.

**Table 3:** Characteristics of duration, disability, and work capacity in pPDPH

Variables	Total *N* = 179	Confirmed *N* = 82	Unconfirmed *N* = 97
Duration of pPDPH	***N (%)***	***N (%)***	***N (%)***
3–6 months	8 (4.5)	4 (4.1)	4 (4.1)
6–12 months	39 (21.8)	15 (18.3)	24 (24.7)
1–3 years	75 (41.9)	29 (35.4)	46 (47.4)
3–5 years	35 (19.6)	24 (29.3)	11 (11.3)
More than 5 years	22 (12.3)	11 (13.4)	11 (11.3)
HDI total score Mean (SD)	49.8 (16.4)	53.8 (18.1)	46.5 (16.5)
Mild Disability (< 35 points)	57 (31.8%)	39 (47.6%)	18 (18.6%)
Moderate Disability (35–60 points)	46 (25.7%)	12 (14.6%)	34 (35.1%)
Severe Disability (> 60 points)	76 (42.5%)	31 (37.8%)	45 (46.3%)
HDI– Emotional Mean (SD)	27.9 (7.9)	28.7 (8.6)	26.2 (7.9)
HDI– Functional Mean (SD)	22.6 (9.8)	25.3 (9.9)	20.3 (9.3)
Positional Headaches			
No	81 (45.3)	45 (54.9)	36 (37.1)
Yes	98 (54.7)	37 (45.1)	61 (62.9)
Location of Headache			
Back Head/Neckregion	90 (50.3)	54 (65.8)	36 (37.1)
Whole Head	58 (32.4)	20 (24.4)	38 (39.2)
Neck Region	23 (12.8)	8 (9.8)	15 (15.4)
Forehead/Temples	8 (4.5)	0	8 (8.3)
Ability to be in an Upright Position			
Not at all	29 (16.2)	16 (19.5)	13 (13.4)
Less than 1 h	50 (27.9)	15 (18.3)	35 (36.1)
2–4 h	29 (16.2)	9 (11.0)	20 (20.6)
Most of the day	16 (8.9)	6 (7.3)	10 (10.3)
Whole day	55 (30.7)	36 (43.9)	19 (19.6)
Sick leave due to pPDPH			
3–6 Months	14 (7.8)	4 (4.9)	10 (10.3)
6–12 Months	43 (24.0)	26 (31.7)	17 (17.5)
Over 1 year	64 (35.8)	26 (31.7)	38 (39.2)
Over 2 years	6 (3.4)	4 (4.9)	2 (2.1)
Unable to work	52 (29.1)	22 (26.8)	30 (30.9)
Current Work Capacity			
Part-time (10–20 h/week)	106 (59.2)	45 (54.9)	61 (62.9)
Full-time (30–40 h/week)	21 (11.7)	14 (17.1)	7 (7.2)
Changed Job Due to pPDPH	2 (1.1)	1 (1.2)	1 (1.0)
Unable to work	50 (27.9)	22 (26.8)	28 (28.9)
Epidural Bloodpatch			
Mean (SD) min-max	3.15 (1.7) 1–9	3.12 (1.9) 1–9	3.2 (1.6) 1–8
Surgery			
Yes	41 (22.9)	41 (50)	0
No	138 ( 77.1)	41 (50)	97 (100)
Explorativ surgery	29 (16.2)	29 (35.5)	0

Note: HDI = headache disability inventory

### Impact of pPDPH on Psychological Well-being, relationship and childcare

The psychological impact was substantial, with over 80% of the entire group reporting depression. Among confirmed cases, 73.2% reported depression, while it was higher at 91.8% in the unconfirmed group. Both confirmed and unconfirmed cases showed high rates of anxiety, approximately 97.6% and 99.0%, respectively, and nearly 90% reported high stress levels in both groups. More than 60% of the total group felt traumatized, with 45.1% in the confirmed group and 78.4% in the unconfirmed group. The effect of pPDPH on personal relationships was significant, with more than half of the confirmed group facing difficulties, higher than the ‘Unconfirmed’ group at nearly 40%. Separation or divorce due to pPDPH was reported by 5.0% in the confirmed group and 19.0% in the unconfirmed group. In the entire group, more than 10% were unable to provide child care, while a similar percentage could only do so for less than an hour, with only a small fraction capable of full-day childcare. Detailed data are provided in Table 4.

**Table 4:** Impact of pPDPH on Psychological Well-being, Relationship and Childcare

Variable	Total *N* = 179	Confirmed *N* = 82	Unconfirmed *N* = 97
DASS21 Score Depression			
No Depression	30 (16.8)	22 (26.8)	8 (8.2)
Depression	149 (83.2)	60 (73.2)	89 (91.8)
DASS21 Score Anxiety			
No Anxiety	3 (1.7)	2 (2.4)	1 (1.0)
Anxiety	176 (98.3)	80 (97.6)	96 (99.0)
DASS21 Score Stress			
No Stress	21 (11.7)	11 (13.4)	10 (10.3)
Stress	158 (88.3)	71 (86.6)	87 (89.7)
Trauma: Do you believe that pPDPH may have triggered a trauma or emotional distress?			
No Negative Impact	4 (2.2)	1 (1.2)	3 (3.1)
I’m uncertain about trauma, but I feel uncomfortable	24 (13.4)	16 (19.5)	8 (8.2)
Feel Traumatized	113 (63.1)	37 (45.1)	76 (78.4)
Emotionally Distressed	38 (21.2)	28 (34.1)	10 (10.3)
Change or difficulties in your relationship			
Problems Worked Through	41 (29.5)	14 (23.3)	27 (34.2)
Significant Difficulties Struggling	65 (46.8)	34 (56.7)	31 (39.2)
Separation/Divorce Due to pPDPH	18 (12.9)	3 (5.0)	15 (19.0)
No Impact on Relationship	7 (5.0)	4 (6.7)	3 (3.8)
Prefer Not to Answer	8 (5.8)	5 (8.3)	3 (3.8)
Ability to take care of children			
Not at all	20 (23.3)	7 (21.2)	13 (24.5)
Under 1 h	23 (26.7)	7 (21.2)	16 (30.2)
2–4 h	17 (19.8)	7 (21.2)	10 (18.9)
4–6 h	15 (17.4)	6 (18.2)	9 (17.0)
Whole day	11 (12.8)	6 (18.2)	5 (9.4)
Support for Household/Childcare			
No Support	55 (30.7)	23 (28.0)	32 (33.0)
Support from Spouse/Partner	75 (41.9)	36 (43.9)	39 (40.2)
Support from Grandparents	24 (13.4)	10 (12.2)	14 (14.4)
Support from Friends	9 (5.0)	7 (8.5)	2 (2.1)
Institutional/State Care	16 (8.9)	6 (7.3)	10 (10.3)

Note: DASS21 = Depression Anxiety Stress Scale-21

### Associations between epidural blood patch procedures, surgical interventions, and clinical variables in PDPH

Patients with mild to moderate headache severity, those without orthostatic headaches, and individuals with a mild to moderate HDI are significantly more likely to be in the unconfirmed pPDPH group, with adjusted odds ratios (AORs) of 3.7, 4.2, and 3.2 respectively, indicating a higher likelihood compared to the confirmed reference group. Being able to work part-time to full-time is associated with almost three time’s higher likelihood of being in the unconfirmed group compared to those unable to work due to pPDPH. Variables such as DASS 21 scores for depression and stress, and trauma history, did not show a statistically significant association with pPDPH confirmation status. Moreover, the results from the generalized linear model suggest that none of the included variables significantly impacted the number of EBP administered, covering aspects like the DASS-21 scores for depression and stress, the presence of positional headaches, trauma, the degree of disability, and the headache category.

### Headache characteristics and others symptoms

All participants reported experiencing headaches, indicating a universal prevalence of this symptom in the study population. Furthermore, the ‘back of the head/neck area’ emerged as the predominant location for headaches with 63.1% of the reports. Headaches encompassing the entire head were the second most commonly reported, accounting for 32.4%, while the ‘temples’ and ‘forehead/temples’ areas were comparatively rare. The severity of the headaches varied, with almost 18% reporting extreme headaches, while approximately one-third experienced severe or moderately severe headaches, and nearly 15% described their headaches as mild. A significant majority (60.3%) consistently experienced symptoms when transitioning to an upright position. Regarding the time until symptom onset or deterioration while in an upright position, about one-fifth experienced it immediately, while approximately 9% reported onset or worsening after 30 min, one hour, or several hours. Notably, almost 40% of the respondents found the question not applicable to their situation. About one-quarter reported significant symptom relief when lying down, but a considerable portion also found the question of alleviation not applicable, suggesting persistent symptoms. Other range of additional symptoms, including nausea, dizziness, pulsating and non-pulsating tinnitus, balance disturbances, sensitivity to light and sound, and pain between the shoulder blades, as detailed in (Appendix Table 3).

## Discussion

Our study underscores the profound and enduring impact of pPDPH on individuals’ well-being. Our data indicate that PDPH can be a long-lasting condition, with some cases enduring for years. These observations challenge the existing definition in the International Classification of Headache Disorders (ICHD-3), which categorizes PDPH as a condition that ‘remits spontaneously within 2 weeks, or after sealing of the leak with an EBP. Our findings, in conjunction with a number of retrospective studies [7, 10, 19, 25, 26], raise questions about this characterization, indicating that PDPH might not always be a self-limiting headache and call for a re-evaluation of PDPH’s definition to better reflect its potential for long-term impact. Additionally, echoing findings from other studies [27–30], our research revealed that only about one third of participants displayed the classic postural component of the headache detailed in the ICHD-3 criteria, where headaches intensify within 15 minutes of sitting or standing and lessen upon lying down. This notable deviation calls for an urgent and thorough review of the current diagnostic guidelines for PDPH, suggesting updates that could deepen our grasp of this condition’s full spectrum and its impact on patients’ lives.

Interestingly despite the relatively common occurrence of headaches following lumbar punctures [10] (nearly half of our group reported lumbar puncture as a trigger), the process leading to an official diagnosis of PDPH is often intricate and not always straightforward. According to the responses of the participants in the study, only 8% reported receiving an official diagnosis of PDPH within 24 h. Slightly over 10% indicated that they were diagnosed within a week. However, the data reveals a significant delay in diagnosis for the majority: nearly 75% of the participants were suspected to have PDPH within three months. Notably, more than half of the respondents stated that they receive a definitive diagnosis of PDPH after more than three months. This substantial delay in diagnosis may contribute to the perceived inefficacy of prevailing therapeutic modalities, potentially stemming from incomplete resolution of the dural tear or the persistent central sensitization of the pain pathway, as initially postulated.

The persistence of symptoms in PDPH is underscored by the diverse degrees of disability reported by participants, with one third experiencing mild, a quarter experiencing moderate, and over 40% experiencing severe disability. Moreover, this online cohort shows high rates of mental health concerns, with almost 90% of participants struggling with symptoms related to depression, anxiety, and stress. These disabilities stem not only from the severe headaches but also from other debilitating symptoms like nausea, dizziness, tinnitus, balance disturbances, and sensitivity to light and sound (Table 5). These symptoms may limit activities so severely that many patients are unable to perform even the most rudimentary activities of their daily lives. Our data underscore the complexity of these conditions, showing that nearly one third of the confirmed group experienced pPDPH symptoms for an extended period of 3–5 years, in contrast to only about 11% in the unconfirmed group. Contrarily, we observe that nearly 40% of the confirmed group can maintain an upright position throughout the day, compared to only about 20% in the unconfirmed group. However, almost half of the participants in both groups struggle to remain upright for longer than an hour, indicating significant impairment. The exact reasons behind these observations remain somewhat elusive. A plausible explanation for the prolonged duration of headaches in confirmed cases might relate to the characteristics of the CSF leak. It is conceivable that larger leaks, more readily identifiable through imaging, pose greater challenges in achieving effective closure with standard interventions like the EBP, potentially necessitating multiple attempts and thus prolonging symptomatology. This difficulty in managing pPDPH effectively is further evidenced by the severe limitations it imposes on daily activities and work. Nearly one-third of affected individuals have been unable to return to work since the onset of their symptoms, and almost 60% are only able to work part-time. A small but notable fraction of the cohort has even had to change their job due to the debilitating effects of pPDPH. These findings emphasize the need for a deeper understanding of pPDPH and the development of more effective management strategies to mitigate its long-term consequences.

**Table 5:** Associations between Diagnostic Status (Confirmed/Unconfirmed PDPH) and the Number of Epidural Blood Patches with Clinical Variables

Variables	AOR (95% CI)	*p*-value
	Confirmed* ( *n* ** = 42) vs. Unconfirmed ( ** *n* ** = 96)**	
**Headache Severity**		
Severe to very severe	Reference	0.006
Mild to moderate	3.7 ( 1.4–9.5)	
**Orthostatic Headache**		
Yes	Reference	0.008
No	4.2 ( 1.4–12.1)	
**Disability Index**		
Severe to complete	Reference	0.006
Mild to moderate	3.2 (1.3–7.5)	
**Workability**		
Not possible due to pPDPH	Reference	0.012
Part-time to full-time	2.9 (1.2–6.6)	
**Dass 21 Depression**		
Yes	Reference	0.989
No	1.1 (0.3–3.8)	
**Dass 21 Stress**		
Yes	Reference	0.318
No	2.2 (0.4–11.1)	
**Trauma**		
Yes	Reference	0.212
No	0.4 (0.2–1.1)	

Dependent Variable: Confirmed PDPH (*n* = 42) vs. Unconfirmed PDPH (*n* = 96) *Note: The Confirmed PDPH group are used as the reference categorie. AOR = Adjusted Odds Ratio = Covariates included in the regressions were age, gender, duration of postdural puncture headache, presence of headaches prior to the onset of postdural puncture headache, and the number of epidural bloodpatch

Our findings also reveal that PDPH severely limits the ability of individuals to perform caregiving duties, with over 10% of respondents unable to care for their children and a similar proportion only able to do so for less than an hour per day. This highlights the extensive daily life limitations experienced by those affected. Studies suggests that unintended dural puncture associated with epidural labor analgesia, can lead to chronic conditions including headaches, backaches, postpartum depression, post-traumatic stress disorder, and difficulties with breastfeeding [20, 25]. The implications of these findings are profound, extending beyond physical health to encompass emotional and psychological distress, and can significantly impact financial stability and self-esteem, potentially leading to increased stress and psychological issues [31]. This complex situation urgently calls for heightened awareness and the creation of more effective management strategies to tackle the extensive implications of pPDPH. Additionally, it is crucial to incorporate the risk of chronic headache into the informed consent process for all neuraxial procedures, ensuring patients are fully aware of potential long-term outcomes. Further, our analysis reveals a pattern where individuals with unconfirmed PDPH are associated with somewhat milder physical symptoms compared to those with confirmed PDPH, which could be attributable to a smaller or slower CSF leak. Despite these associations, the extent of psychological distress is comparable between both groups, suggesting that concerns, anxiety, and stress about overall health might have an equal impact on psychological well-being as the severity of physical symptoms.

While our study focuses on pPDPH, it is important to note that prior research on the HRQOL and disability in this specific condition has been limited. Until now, studies evaluating HRQOL and disability in conditions with similar symptomatology, such as SIH, have been more prevalent. For instance, the study by Liaw et al. [32] on individuals with SIH provides notable parallels. Their study revealed significantly lower SF-36 scores, indicating substantial physical health limitations, pain, compromised physical functioning, and reduced energy levels, along with almost half experiencing moderate depression and a significant portion having moderate anxiety. The high prevalence of suicidal thoughts and behaviors in their cohort, with over half expressing a desire to be deceased and nearly one in four reporting past suicidal tendencies, underscores the severe mental health impact of this condition. Transitioning from this, the recent study by Volz et al. [33] provides an essential expansion of our understanding in this field. Their research, focusing on SIH, uncovers that symptoms of depression, anxiety, and stress are not only prevalent but also show significant improvement post-treatment. This revelation challenges the prior notion that mental health issues are not primary symptoms in SIH, suggesting instead that such depressive symptoms might be more a reaction to the physical illness rather than independent psychiatric conditions. In parallel, our study sheds light on the complex psychological challenges faced by pPDPH patients. Over a third of participants report major issues in their relationships, with 10% leading to separations or divorces due to the strain of the illness. Additionally, a concerning 63% of those with pPDPH feel a sense of trauma, highlighting the serious mental health concerns associated with the condition. These findings underline the importance of comprehensive care that includes psychological support for individuals dealing with pPDPH.

Further, in the field of SIH treatment, recent studies have provided insightful findings. In a study by Jesse et al. [34], most patients with confirmed SIH due to spinal CSF leaks remained impaired in HRQoL even after surgical treatment. This is echoed by Brinjikji et al. [17], who found a 95% treatment response in SIH patients post-transvenous embolization, yet not all achieved complete symptom resolution. Volz et al.‘s study [33] also showed significant long-term improvements post-surgical closure of spinal CSF leaks, but a quarter of patients continued to face long-term impairments. In addition, the study by Cheema et al. [35], involving SIH patients, found that a significant proportion of participants suffered considerable impacts on their work life due to SIH. A quarter of the patients lost their jobs, and 60% had to alter their work duties. This study also reported a negative impact on quality of life, as measured by the EQ-5D-5 L questionnaire. The authors concluded that SIH is a highly disabling condition that affects multiple aspects of life, which is in line with our findings. A further explanation could be that other types of headaches, such as migraines or tension headaches, as well as post-therapeutic rebound hypertension headache, might be contributing factors [33]. In the literature, the incidence of rebound headache is reported to be around 27%, but its exact pathogenesis remains not fully understood [36]. This complexity suggests the need for continued research into the underlying causes and potential treatments for these persisting symptoms. Building on these insights into the long-term impact of PDPH, we find a resonating theme in the work of Zhang et al. [7] concerning PDPH patient follow-up. We are in complete alignment with Zhang and colleagues, strongly endorsing their research that highlights a crucial aspect often overlooked: despite PDPH being viewed as self-limiting, about 91% of patients with chronic headaches after EBP were asymptomatic upon hospital discharge [37]. This significant finding reinforces our shared viewpoint on the necessity of thorough post-discharge monitoring and specialist referral for patients experiencing pPDPH.

There are both strengths and limitations to this online survey. One of the primary strengths of this study is its novelty, as it represents, to our knowledge, the first study focusing on pPDPH with regard to its impact on social life, work ability, and quality of life. Furthermore, the study’s collaborative approach, involving individuals who have personal experience with pPDPH, adds a unique and valuable perspective. This patient-centered approach ensures that the survey questions reflect the most pertinent concerns of those affected, providing insights that are deeply rooted in the actual experiences of pPDPH patients. The involvement of an author who is himself a sufferer of pPDPH further enhances the relevance and authenticity of the study, ensuring that it resonates with the patient community and addresses the condition in a comprehensive manner. Additionally, the study calls for a reevaluation of the ICHD-3 definition of PDPH, particularly in terms of its criteria for spontaneous remission within two weeks or after an EBP and that not all affected individuals exhibit a postural component. Our findings, highlighting the condition’s prolonged duration and the persistence of symptoms and challenges in daily life despite treatment, emphasize the need for an updated definition that accurately reflects the long-term impacts observed. This recommendation for a revised definition enhances our study’s significance in encouraging advancements in the classification and care of headache patients.

However, the study also has limitations that warrant consideration. One such limitation is the potential for recall bias, especially given the varying degrees of severity and disability among participants at different stages of their condition. This bias could impact the accuracy of participants’ recollections regarding their treatment and diagnostic experiences. Recall bias is an inherent challenge in retrospective studies and can influence the data, particularly when participants are asked to recall past events over extended periods. Additionally, the method of recruitment, predominantly through online self-help groups, could introduce a selection bias, possibly resulting in a sample that may not be fully representative of the broader pPDPH population. This method might not capture a fully representative cross-section of the pPDPH population, possibly skewing towards those with more severe symptoms or a higher propensity to seek support and information online. Such a bias could influence our findings, suggesting a potential overestimation of the severity and impact of pPDPH. We recognize this limitation and its implications for the generalizability of our results.

## Conclusions

Our study on pPDPH reveals the intricate challenge of managing this condition, which is not always self-limiting as previously expected. Our findings reveal that the burden of pPDPH arises not just from the severity of headaches but also from a spectrum of debilitating symptoms. We observed that confirmed cases typically displayed slightly more severe physical symptoms but the degree of psychological distress remained similar across confirmed and unconfirmed cases, emphasizing the profound emotional effects of pPDPH regardless of diagnostic status. These insights underscore the critical need for a comprehensive treatment strategy that addresses both the physical and psychological aspects of pPDPH, emphasizing the importance of timely diagnosis and effective intervention. Our findings illuminate the chronic and multifaceted nature of pPDPH, highlighting the necessity for insurance policies to encompass a wide range of care services, from acute treatment to ongoing management, such as pain support. These insights could be invaluable to insurance providers in understanding the comprehensive care requirements of pPDPH patients, ensuring that policies are tailored to meet the actual, long-term needs of those affected.

### Electronic supplementary material

Below is the link to the electronic supplementary material.

Supplementary Material 1

Supplementary Material 2

## Data Availability

No datasets were generated or analysed during the current study.

## Abbreviations

PDPH: Postdural Puncture Headache
pPDPH: Persistent Postdural Puncture Headache
CSF: Cerebrospinal Fluid
EBP: Epidural Blood Patch
IH: Intracranial hypotension
SIH: Spontaneous Intracranial Hypotension
MRI: Magnetic Resonance Imaging
CT: Computed Tomography
HRQoL: Health-Related Quality of Life
DASS-21: Depression Anxiety Stress Scale-21
HDI: Henry Ford Hospital Headache Disability Inventory
