# Supplementary material for: Health-related quality of life, work ability and disability among individuals with persistent post-dural puncture headache
Source: J Headache Pain. 2024 Apr 24;25(1):64. doi: 10.1186/s10194-024-01765-8 (PMC11040840; doi:10.1186/s10194-024-01765-8)
Supplement: Supplementary file 1 — Supplementary Material 1 [file 10194_2024_1765_MOESM1_ESM.docx]

**Appendix A**

**Table 1. Other medical Conditions**

| Medical Condition | Frequency | Percent |
| --- | --- | --- |
| Ehlers-Danlos syndromes | 15 | 8.4 |
| Postural orthostatic tachycardia syndrome | 10 | 5.6 |
| Multiple sclerosis | 9 | 5.0 |
| Stroke | 6 | 3.4 |
| Cancer | 5 | 2.8 |
| Chiari Malformation | 5 | 2.8 |
| Liver disease | 3 | 1.7 |
| Parkinson's disease | 3 | 1.7 |
| Diabetes | 2 | 1.1 |
| Syringomyelia | 1 | 0.6 |

**Table 2. Participants by countries**

| Country | Frequency | Percent |
| --- | --- | --- |
| Australia | 11 | 6.1 |
| Austria | 4 | 2.2 |
| Belgium | 1 | 0.6 |
| Canada | 6 | 3.4 |
| France | 1 | 0.6 |
| Germany | 52 | 29.1 |
| Italy | 2 | 1.1 |
| Norway | 1 | 0.6 |
| Poland | 2 | 1.1 |
| Portugal | 5 | 2.8 |
| Spain | 6 | 3.3 |
| Sweden | 5 | 2.8 |
| Switzerland | 1 | 0.6 |
| United Kingdom | 50 | 28.1 |
| United States | 32 | 18.0 |

**Table 3. Symptoms in pPDPH**

| Category | Headache | Nausea | | | Vertigo | | Pulsating Tinnitus | | | Nonpulsating Tinnitus | | Other Hearing Symptoms | | | Sense of Imbalance | | Sensitivity to Light | | | Sensitivity to Sound | | | Pain between Shoulder Blades |
| --- | --- | --- | --- | --- | --- | --- | --- | --- | --- | --- | --- | --- | --- | --- | --- | --- | --- | --- | --- | --- | --- | --- | --- |
|  | Frequency (%) | Frequency (%) | | | Frequency (%) | | Frequency  (%) | | | Frequency  (%) | | Frequency  (%) | | | Frequency  (%) | | Frequency  (%) | | | Frequency  (%) | | | Frequency  (%) |
| Yes | 179 (100) | 79 (44.1) | | | 89 (49.7%) | | 78 (43.6) | | | 94 (53.1) | | 114 (63.7) | | | 100 (55.9) | | 81 (45.3) | | | 82 (45.8) | | | 102 (57.0) |
| No | 0 | 100 (55.9) | | | 90 (50.3) | | 101 (56.4) | | | 85 (46.9) | | 65 (36.3) | | | 79 (44.1) | | 98 (54.7) | | | 97 (54.2) | | | 77 (43.0) |
| Severity |  |  | | |  | |  | | |  | |  | | |  | |  | | |  | | |  |
| Extremely Severe | 32 (17.9) | 9 (5.0) | | | 16 (8.9) | | 10 (5.6) | | | 25 (14.0) | | 29 (16.2) | | | 22 (12.3) | | 5 (2.8) | | | 14 (7.8) | | | 17 (9.5) |
| Severe | 61 (34.1) | 24 (13.4) | | | 21 (11.7) | | 18 (10.1) | | | 11 (6.1) | | 24 (13.4) | | | 23 (12.8) | | 19 (1.6) | | | 25 (14.0) | | | 37 (20.7) |
| Moderate | 60 (33.5) | 26 (14.5) | | | 43 (24.0) | | 35 (19.6) | | | 34 (19.0) | | 38 (21.2) | | | 42 (23.5) | | 38 (21.2) | | | 27 (15.1) | | | 35 (19.6) |
| Mild | 26 (14.5) | 20 (11.2) | | | 9 (5.0) | | 15 (8.4) | | | 24 (13.4) | | 23 (12.8) | | | 13 (7.3) | | 19 (10.6) | | | 13 (7.3) | | | 13 (7.3) |
| None | 0 | 100 (55.9) | | | 90 (50.3) | | 101 (56.4) | | | 85 (47.5) | | 65 (36.3) | | | 79 (44.1) | | 98 (54.7) | | | 100 (55.9) | | | 77 (43.0) |
| Presence of symptoms to transition to upright position | | | |  | | | |  | | |  | | |  | | | |  | | |  | | |
| Always | 108 (60.3) | 11 (6.1) | | | 16 (8.9) | | 21 (11.7) | | | 34 (19.0) | | 49 (27.4) | | | 19 (10.6) | | 21 (11.7) | | | 21 (11.7) | | | 45 (25.1) |
| Most of the time | 12 (6.7) | 5 (2.8) | | | 17 (9.5) | | 17 (9.5) | | | 10 (5.6) | | 21 (11.7) | | | 16 (8.9) | | 10 (5.6) | | | 38 (21.2) | | | 5 (2.8) |
| Often | 16 (8.9) | 33 (18.4) | | | 16 (8.9) | | 22 (12.3) | | | 10 (5.6) | | 13 (7.3) | | | 23 (12.8) | | 21 (11.7) | | | 18 (10.1) | | | 20 (11.2) |
| Sometimes | 24 (13.4) | 25 (14.0) | | | 28 (15.6) | | 17 (9.5) | | | 20 (11.2) | | 23 (12.8) | | | 36 (20.1) | | 15 (8.4) | | | 18 (10.1) | | | 21 (11.7) |
| Rarely | 19 (10.6) | 5 (2.8) | | | 12 (6.7) | | 1 (0.6) | | | 20 (11.2) | | 8 (4.5) | | | 6 (3.4) | | 14 (7.8) | | | 5 (2.8) | | | 11 (6.1) |
| Never | 0 | 100 (55.9) | | | 90 (50.3) | | 101 (56.4) | | | 85 (47.5) | | 65 (36.3) | | | 79 (44.1) | | 98 (54.7) | | | 97 (54.2) | | | 77 (43.0) |
| Time until onset/deterioration | | |  | | |  | | |  | | | |  | | |  | | |  | | |  | |
| Immediately | 39 (21.8) | 18 (22.8) | | | 39 (43.8) | | 20 (25.6) | | | 10 (10.6) | | 39 (34.2) | | | 44 (44.0) | | 6 (7.4) | | | 18 (22.0) | | | 16 (15.7) |
| After 15 minutes | 22 (12.3) | 11 (13.9) | | | 22 (24.7) | | 25 (32.1) | | | 20 (21.3) | | 24 (21.1) | | | 16 (16.0) | | 5 (6.2) | | | 10 (12.2) | | | 18 (17.6) |
| After 30 minutes | 15 (8.4) | 14 (17.7) | | | 5 (5.6) | | 5 (6.4) | | | - | | 8 (7.0) | | | 10 (10.0) | | 6 (7.4) | | | 8 (9.8) | | | 8 (7.8) |
| After 1 hour | 16 (8.9) | 7 (8.9) | | | 2 (2.2) | | 2 (2.6) | | | 3 (3.2) | | 13 (11.4) | | | 13 (13.0) | | 3 (3.7) | | | 11 (13.4) | | | 14 (13.7) |
| After several hours | 16 (8.9) | 8 (10.1) | | | 16 (18.0) | | 7 (9.0) | | | 13 (13.8) | | 10 (8.8) | | | 7 (7.0) | | 17 (21.0) | | | 10 (12.2) | | | 26 (25.5) |
| Not applicable | 71 (39.7) | 21 (26.6) | | | 5 (5.6) | | 19 (24.4) | | | 48 (51.1) | | 20 (17.5) | | | 10 (10.0) | | 44 (54.3) | | | 25 (30.5) | | | 20 (19.6) |
| Alleviation |  |  | | |  | |  | | |  | |  | | |  | |  | | |  | | |  |
| Completely | 3 (1.7) | 2 (2.5) | | | 19 (21.3) | | 7 (8.9) | | | 5 (5.32) | | 22 (19.3) | | | 49 (49.0) | | 5 (6.1) | | | 3 (3.6) | | | 9 (8.8) |
| Much Better | 41 ( 22.9) | 28 (35.4) | | | 45 (50.5) | | 7 (8.9) | | | 8 (8.51) | | 28 (24.5) | | | 39 (39.0) | | 15 (18.5) | | | 22 (26.8) | | | 21 (20.5) |
| Somewhat Better | 64 (35.8) | 25 (31.6) | | | 18 (20.2) | | 45 (57.6) | | | 21 (22.3) | | 47 (41.2) | | | 2 (2.0) | | 24 (29.6) | | | 33 (40.2) | | | 56 (54.9) |
| Not Applicable | 71 ( 39.7) | 24 (30.3) | | | 7 (7.8) | | 19 (24.3) | | | 60 (63.8) | | 17 (14.9) | | | 10 (10.) | | 37 (45.6) | | | 24 (29.2) | | | 16 (15.6) |
