# Supplementary material for: Health-related quality of life, work ability and disability among individuals with persistent post-dural puncture headache
Source: J Headache Pain. 2024 Apr 24;25(1):64. doi: 10.1186/s10194-024-01765-8 (PMC11040840; doi:10.1186/s10194-024-01765-8)
Supplement: Supplementary file 2 — Supplementary Material 2 [file 10194_2024_1765_MOESM2_ESM.docx]

**Appendix B. Checklist for Reporting Results of Internet E-Surveys (CHERRIES) for PPDPH Survey.**

| **Item Category** | **Checklist Item** | **Explanation** |
| --- | --- | --- |
| **Design** | Survey Design | Cross-sectional online survey focused on the experiences of individuals with PPDPH. Conducted globally, the survey consisted of 5 sections: Section 1 – Demographics and Characteristics of PPDPH, Section 2 - Treatment, Section 3 - Diagnostic, Section 4 - The Depression Anxiety Stress Scale-21, and Section 5 - Hospital Headache Disability Inventory. The survey comprised 106 questions across 20 pages, Survey questions are listed in Appendix B. |
| **IRB Approval and Informed Consent Process** | IRB Approval | Ehical approval obtained from the Medical University of Vienna, approval number 1180/2023. |
|  | Informed Consent | An online consent process where participants clicked “Agree” to signify understanding and meeting inclusion criteria. Here is the text for consent “Dear Sir/Madam, We cordially invite you to participate in our online survey. Participation in this anonymous study is voluntary, and you may withdraw from it at any time without providing a reason. Refusing to participate or withdrawing early from the study will not have any adverse consequences for you. The purpose of the survey is to better understand the challenges and needs of individuals with PPDPH. The development of this type of headache can lead to significant impairments, including lost work days, unemployment, inability to work, and reduced quality of life (QOL). As a participant, you will have the opportunity to share your own perspectives and experiences with PPDPH and other related symptoms. This study is conducted on behalf of the Medical University of Vienna, Center for Public Health at the Institute for Social and Preventive Medicine. Your participation in the study is voluntary, and your data will be treated as anonymous and confidential. No personal information, such as IP address, name, or email address, will be collected or stored. The study is expected to take approximately 30 minutes to complete. By clicking on "Next" at the bottom right, you automatically agree to participate in this study. We aim to have at least 100 participants take part in this study. If you have any further questions or concerns, please do not hesitate to contact the project lead, Ali Kapan, at the Medical University of Vienna, Center for Public Health, Institute for Social and Preventive Medicine”. |
| **Data Protection** | Data Protection | Data was collected using the SoSci Survey platform, known for its strong emphasis on data security and privacy. The platform ensures that all data transfer is encrypted using SSL (HTTPS) protocols. Additionally, data storage and processing conform to the stringent regulations of the GDPR, as the servers are located in Germany. The entire data analysis process was conducted on encrypted devices to further safeguard the integrity and confidentiality of the data collected. No personally identifiable information, such as names or IP addresses, was collected, ensuring participant anonymity and privacy. |
| **Development and Pretesting** | Development and Testing | The development of the survey was a collaborative effort, involving clinical researchers, PPDPH patients, and experts in the field of headaches. Notably, one of the authors, a patient with personal experience of chronic Postdural puncture Headache, contributed significantly to the survey design, ensuring a patient-centered approach and relevance. Another author, leading the headache department at the AKH Vienna, brought extensive clinical expertise to the project. This combination of personal experience and professional expertise was instrumental in formulating questions that accurately capture the complexities of PPDPH. The survey was pilot-tested to assess its technical functionality and the clarity of its questions, ensuring it effectively addressed the key research objectives. |
| **Recruitment Process and Sample Description** | Open vs. Closed Survey | This was a closed survey, targeting a specific demographic – adults diagnosed or suspected with PPDPH. |
|  | Contact Mode | The survey was promoted through social media platforms of specific organizations related to PPDPH reaching an audience of over 17,000 followers. This approach utilized the unique communities and networks within each group, ensuring a more focused and effective recruitment. The survey link, <https://www.soscisurvey.de/csfleak/>, was shared on these platforms, and group administrators played a key role in monitoring and facilitating the survey's distribution, thereby enhancing its visibility and participation rate among relevant individuals. |
|  | Advertising the Survey | Prior to announcing the survey on social media, the research team sought permission from administrators of relevant Facebook groups. Upon receiving approval, a detailed survey advertisement was posted in each group. This advertisement included comprehensive information about the study, highlighting its purpose and voluntary nature. The post also contained a direct link to the survey and information about informed consent. This process ensured ethical conduct and respect for the group's norms, while effectively reaching the target audience for the survey. |
| **Survey Administration** | Web/E-mail | The survey was hosted online, with a specific URL (<https://www.soscisurvey.de/csfleak/>). The interface was customized for user-friendliness and clarity. |
|  | Context | No specific website was used; the survey was directly accessible via the provided URL. |
|  | Mandatory/Voluntary | Participation was voluntary, with a detailed explanation and consent process at the beginning. |
|  | Incentives | No incentives were offered; participation was entirely voluntary and aimed at academic research. |
|  | Time/Date | The survey was active from April 19 to June 19, 2023. To ensure continuous engagement, weekly reminders about the survey were periodically posted on these platforms. |
|  | Randomization of Items | Questions were presented in a fixed order for each participant. |
|  | Adaptive Questioning | Adaptive questioning principles were used to number and handle the complexity of the questions. |
|  | Number of Items | The survey consisted of 5 sections: Section 1 – Demographics and Characteristics of PPDPH, Section 2 - Treatment, Section 3 - Diagnostic, Section 4 - The Depression Anxiety Stress Scale-21, and Section 5 - Hospital Headache Disability Inventory. The survey comprised 106 questions across 20 pages. |
|  | Number of Screens | Survey distributed over 20 pages. |
|  | Completeness Check | The survey incorporated both mandatory and optional questions. Clinical characteristic questions required a response, ensuring critical data were thoroughly collected. In contrast, standardized questionnaires allowed skipping questions, offering flexibility to participants. Additionally, all questions featured "not applicable" or "don't know" options, accommodating varied respondent experiences and maintaining data integrity. |
|  | Review step | Respondents were able to move between pages to change responses. However, once they submitted the survey the responses were final. |
| **Response Rates** | Unique Site Visitor | Total of 1448 clicks, including unintentional clicks. |
|  | View Rate | Not calculated. |
|  | Participation Rate | A total of 347 individuals consented to participate in the survey. |
|  | Completion Rate | 179 out of 347 participants completed the survey. |
| **Preventing Multiple Entries** | Cookies and IP Check | No use of cookies; IP addresses were not collected for duplicate entry checks. |
|  | Log File Analysis | Not applicable. |
|  | Registration | Each participant accessed the survey via unique online link. |
| **Analysis** | Handling Incomplete Questionnaires | Only fully completed surveys were analyzed. |
|  | Questionnaires with Atypical Timestamp | Not applicable. |
|  | Statistical Correction | Statistical Correction: No specific statistical correction methods were employed in the analysis of our survey data. Given the descriptive nature of our study, our focus was on presenting straightforward, unweighted frequencies and percentages to accurately reflect the responses received. Our approach was to provide a clear and direct representation of the data as collected, without applying additional statistical adjustments or weighting. This approach aligns with our objective to offer a descriptive overview of the participants' responses, rather than inferential or predictive analyses. As a result, our data analysis primarily involved basic descriptive statistics, ensuring a transparent and direct presentation of the findings. |
